# Occupational exposure to respirable crystalline silica and chronic non-malignant renal disease: systematic review and meta-analysis

**DOI:** 10.1007/s00420-017-1219-x

**Published:** 2017-04-13

**Authors:** Matthias Möhner, Anne Pohrt, Johannes Gellissen

**Affiliations:** Division of Work and Health, Federal Institute for Occupational Safety and Health, Nöldnerstr. 40/42, 10317 Berlin, Germany

**Keywords:** Respirable silica, Chronic renal disease, Review, Meta-analysis, SMR

## Abstract

**Background:**

While occupational exposure to respirable silica is known to lead to lung disease, most notably silicosis, its association with chronic kidney disease is unclear.

**Objectives:**

This review explores the association between occupational exposure to respirable silica and chronic non-malignant renal disease such as glomerulonephritis. The evidence has been collected and compiled. Possible sources of bias are thoroughly discussed.

**Methods:**

Cohort studies with silica exposure and case–control studies of renal disease were searched in PubMed until January 2015. Two authors independently abstracted data; any disagreement was resolved by consulting a third reviewer. A meta-analysis was performed to evaluate the association to silica exposure.

**Results:**

A total of 23 cohort and four case–control studies were included in the analysis. The meta-analysis of cohort studies yielded elevated overall SMRs for renal disease. Some studies, however, included dose–response analyses, most of which did not show a positive trend. The approaches and results of the case–control studies were very heterogeneous.

**Conclusions:**

While the studies of cohorts exposed to silica found elevated SMRs for renal disease, no clear evidence of a dose–response relationship emerged. The elevated risk may be attributed to diagnostic and methodological issues. In order to permit a reliable estimation of a possible causal link, exposed cohorts should be monitored for renal disease, as the information from mortality studies is hardly reliable in this field.

**Electronic supplementary material:**

The online version of this article (doi:10.1007/s00420-017-1219-x) contains supplementary material, which is available to authorized users.

## Background

### Silica and occupational disease

Silica (SiO_2_) is the second most abundant mineral in the Earth’s crust. It is found in sand, rock, and soil. Workers particularly exposed to silica include miners, quarrymen, smelters, sandblasters, masons, and ceramic and glass manufacturers. The primary pathway of silica into the human body is through the respiratory organs as dust; the exposure via skin or digestive tract is negligible.

Exposure to silica has long been known to be associated with lung diseases, most notably silicosis. In 2009, the International Agency for Research on Cancer (IARC) classified respirable crystalline silica as a human carcinogen of group 1 (sufficient evidence) (IARC 2012). But correlations have also been observed between exposure to respirable silica and other diseases, like scleroderma, systemic lupus erythematodes, rheumatoid arthritis, or ANCA-associated vasculitis (Gomez-Puerta et al. 2013).

Case studies of non-malignant renal disease in conjunction with silica exposure have motivated case–control studies and occupational cohort studies targeting chronic kidney disease (CKD) and specifically glomerulonephritis (GN). First reviews have been published (Bartsch et al. 2004; Stratta et al. 2001). Moreover, NIOSH reviewed results on risks for renal diseases in its report on quartz dust, but without systematic summary or final evaluation (NIOSH 2002).

### Etiology of renal disease

In advanced stages, chronic renal disease is characterized by concurrent changes in glomeruli, tubuli, blood vessels, and interstices (Gross et al. 1996). However, it usually sets in with very few symptoms, making epidemiologic research on its causes difficult. Globally, the most frequent causes of renal disease that can lead to renal failure are considered to be inflammations and infections.

In Germany, QuaSi-Niere collects data on dialysis and renal transplant patients and has recorded the causes of renal disease since 1997 (Frei and Schober-Halstenberg 2008). Predominant causes are diabetes mellitus and arterial hypertension, which constitute the causes of two-thirds of all chronic renal failures, whereas glomerulonephritis plays only a minor role. The data also show an increase in vascular and diabetic nephropathies probably due to the increasing proportion of older and multimorbid patients. Another cause for renal damage can be medication; besides some antibiotics, nephrotoxic drugs also include non-steroidal analgesics, especially aspirin or phenazone combined with paracetamol, caffeine, codeine, and phenacetin, which can induce analgesic nephropathy after long-term use (Kuhlmann et al. 1998).

Furthermore, several heavy metals damage the kidney by accumulating in the renal cells and blocking metabolic processes, primarily in the tubuli, leading to proteinuria and necrosis of the tubuli. Proteinuria among workers handling cadmium was described for the first time in an alkaline battery plant (Friberg 1950). Cadmium was also identified as the leading cause of the endemic “itai–itai” disease in Japan, which was identified as a sort of osteomalacia combined with proteinuria and glycosuria (Emmerson 1970; Tsuchiya 1969). An overview of cadmium as a nephrotoxin was given by Järup (Järup 2003).

Among the occupational toxins that cause CKD are also mercury (Aymaz et al. 2001; Kazantzis 1970; Miller et al. 2013; Voitzuk et al. 2014) and lead (Ekong et al. 2006; Evans et al. 2010; Lin et al. 2006). But also uranium and arsenic impair the renal function in humans (Brugge and Buchner 2011; Landrigan et al. 1984; Sabath and Robles-Osorio 2012; Tchounwou et al. 2003; Zheng et al. 2015); recent cohort studies among uranium miners and millers support this assumption (Kreuzer et al. 2015; Pinkerton et al. 2004; Schubauer-Berigan et al. 2009).

The nature of a possible association of silica with kidney damage has not been fully explained. Pathologic changes similar to those due to nephrotoxic heavy metals make a direct toxic effect plausible; since the detection of anti-nucleic antibodies, an immunologic process has also been discussed (Bartsch et al. 2004; Marquardt and Schäfer 2003).

### Research question

Therefore, the aim of this review is to systematically explore the available evidence on the association between occupational exposure to respirable silica and the risk of renal damage. Outcomes under consideration are chronic non-malignant renal diseases with a focus on glomerulonephritis.

## Materials and methods

### Systematic search and evaluation

The review is reported following the PRISMA statement (Moher et al. 2009). Relevant studies were selected through a systematic search in MEDLINE for publications from 1987 to Jan 15, 2015 as well as reference tracking and a hand search. We aimed at epidemiological studies on chronic non-malignant renal diseases and, more specifically, glomerulonephritis in conjunction with occupational exposure to respirable silica. Both cohort studies and case–control studies were accepted. Studies investigating cadmium, lead, mercury, uranium, or arsenic exposure were excluded. Furthermore, studies on asbestos exposure were excluded. The first reason for their exclusion was that there are some epidemiological studies showing an elevated risk for renal cancer with respect to asbestos exposure (Enterline et al. 1987; Mattioli et al. 2002; Mellemgaard et al. 1994; Selikoff et al. 1979). Although the corresponding reviews produced contradictory findings (Sali and Boffetta 2000; Smith et al. 1989), an asbestos-related impairment of the kidney cannot be excluded. The second reason was that most studies on workers exposed to silica and asbestos reported results on asbestos-related cancer and respiratory tract diseases only.

Finally, to find all relevant studies, the primary search string was set to

- ((glomerulonephritis OR kidney OR renal OR nephritis) AND (silica* OR quartz OR sand OR “silicon dioxide” OR “silicon compounds”) AND (cohort OR follow-up OR longitudinal OR case–control OR case-referent OR case-cohort OR case/control)) NOT (asbestos OR cadmium OR lead OR mercury OR uranium OR arsenic).

Numerous cohort studies have investigated the association between respirable silica exposure and health outcomes such as silicosis, non-malignant respiratory disease, or lung cancer. These studies frequently reported standardized mortality ratios (SMR) regarding diseases outside of their primary outcomes. Hence, to also find this kind of cohort studies, a secondary search string was set to

- ((silica OR silicosis) AND (cohort OR mortality OR longitudinal OR follow-up) AND (occupation* OR worker* OR miner OR miners)) NOT (asbestos OR cadmium OR lead OR mercury OR uranium OR arsenic).

All references were evaluated by two reviewers (JG, AP) with title and abstract using the following criteria: Only cohort or case–control studies with a human male adult study population with quartz dust exposure were selected. Case studies, surveys, intervention studies, and studies on children or animals or in vitro or in vivo were rejected.

In the next step, full papers were read for selected studies and studies without information on renal disease were discarded. Where several studies on the same cohort existed, the most recent one was selected. Case–control studies were checked for adequate outcomes. They were discarded if the control group contained patients with renal disease.

In many cases, studies were excluded because on closer inspection they did not fulfill the inclusion criteria. Where the decision merited discussion, we documented the reason for exclusion (see Electronic Supplementary Material).

### Meta-analysis

A meta-analysis was conducted for the cohort studies, separately for occupational cohorts and cohorts from workers suffering from silicosis which were reported to a silicosis registry. Standardized mortality ratios were adjusted for silicosis as a competing cause of death (Möhner 2016). Where the number of cases had been extrapolated (Marinaccio et al. 2006; Scarselli et al. 2011), the actual number of cases was used.

Results were compared and checked for heterogeneity. In order to achieve better comparability, similar outcomes were used. The group ICD-9: 580–589 (Nephritis, nephrotic syndrome, and nephrosis) was preferred, but some studies provided only data on the whole ICD-chapter X (Diseases of the genitourinary system).

Statistical analyses were performed using the statistical software package Stata, release 14. The *metan* command was used for meta-analysis (Harris et al. 2008).

## Results

### Search results

**Fig. 1:**
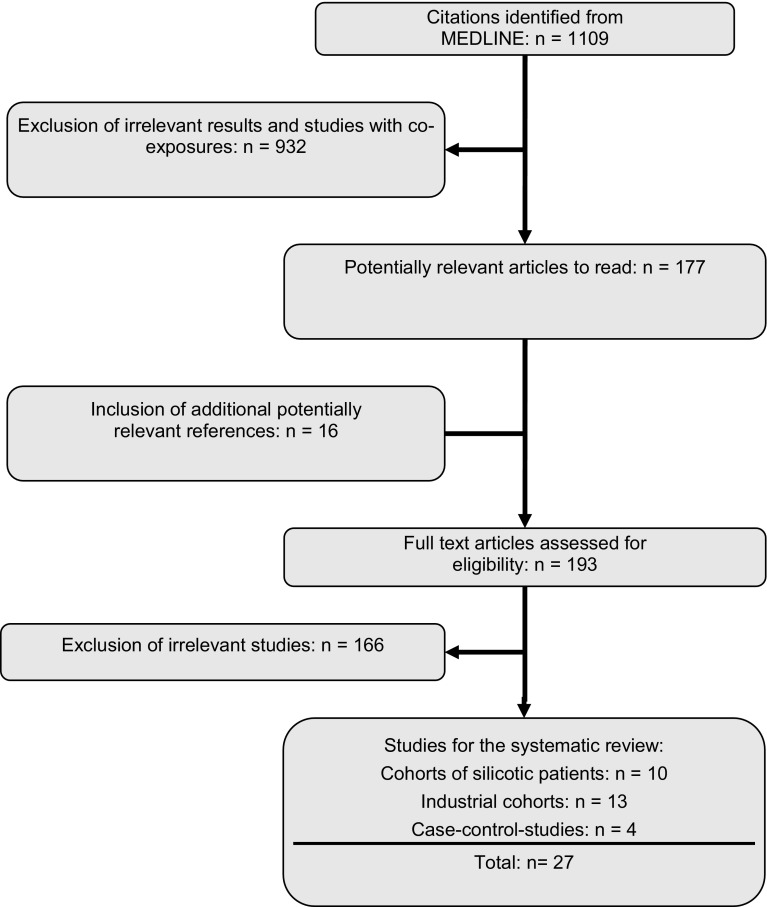
Flow chart of the literature search

The search in PubMed yielded 1109 publications. Figure 1 shows the further stages of the study selection. In the end, the selection and evaluation procedure yielded the following:

- 10 cohort studies from silicosis registries (Amandus et al. 1991; Brown et al. 1997; Ebihara and Kawami 1998; Forastiere et al. 1989; Marinaccio et al. 2006; Ng et al. 1990; Scarselli et al. 2011; Starzynski et al. 1996; Steenland et al. 2002; Yu et al. 2007),
- 13 industry-based cohort studies (Birk et al. 2009; Björ et al. 2013; Brown and Rushton 2005; Checkoway et al. 1997; Cherry et al. 2013; Koskela et al. 1987; McDonald et al. 2005; Morfeld et al. 2005; Olsen et al. 2012; Steenland and Brown 1995; Steenland and Sanderson 2001; Vacek et al. 2011), and
- 4 case–control studies (Calvert et al. 2003; Chiazze et al. 1999; Steenland et al. 1990; Vupputuri et al. 2012). The main characteristics of the included studies are given in Tables 1, 2, 3.

**Table 1:** Industry-based cohort studies

References	Industry sector, country	Cause of death	Cohort size	Period of employment	Follow-up period	Total deaths	No. of observed cases	Effect size (95%-CI)	Loss to follow-up	Exposure estimation; mean exposure time (in years)
Birk et al. (2009)	Porcelain industry, Germany	Renal diseases (ICD-10: N00–N08, N10–N12, N14–N19, N26–N29)	8288	1895–1987	1985–2005	1126	10	1.32 (0.63–2.43)	7%	Decade of hire, hire before or after the end of 1960, ever having worked in the preparation area; 21.5
Björ et al. (2013)	Iron ore mining, Sweden	Genitourinary diseases (ICD-10: N00–N99)	13,000	1923–1998	1952–2006	5185	65	0.96 (0.74–1.23)	n/a	Duration of employment as blue-collar worker; n/a
Brown and Rushton (2005)	Sand industry, UK	Genitourinary diseases	2703	1950–1986	1950–2001	764	9	0.99 (0.45–1.87)	n/a	JEM using samples between 1978 and 2000 and extrapolation back to 1950, n/a
Checkoway et al. (1997)	Diatomaceous earth mining, California, USA	Genitourinary diseases	2342	1942–1987	1942–1994	749	10	1.06 (0.51–1.94)	n/a	JEM using measurements between 1948 and 1988 and extrapolation back to 1942; 5.54
Cherry et al. (2013)	Pottery, Staffordshire, UK	Renal diseases (ICD-9: 581, 582, 583, 585–589) (ICD-10: N03–N05, N18, N19)	5115	1931–1992	1985–2008	1904	14	3.5 (1.91–5.87)	0.6%	Duration of pottery work before 1992, type of pottery work before 1992 for a subgroup of 3506 subjects; 13.8
Koskela et al. (1987)	Granite industry, Finland	Nephritis, Nephrotic syndrome, Nephrosis, (ICD-8: 580-589)	1026	1940–1971	1940–1981	235	1	0.83 (0.02–4.64)	n/a	JEM using RCS samples between 1970 and 1972; 12
McDonald et al. (2005)	Sand industry, USA	Nephritis/nephrosis (ICD-9: 580.0–589.9)	2452	1940–1979	1940–2000	1221	18	2.80 (1.66–4.42)	0.9%	JEM using RCS samples between 1975 and present, interpolation to changes and particle counts from 1946 to 1955; n/a
Morfeld et al. (2005)	Hard coal mining, Germany	Renal diseases (ICD-9: 580–587)	4579	1980–2002	1980–2002	1183	13	1.13 (0.60–1.93)	0.5%	JEM based on measurements 1980–2001; 30.4
Olsen et al. (2012)	Roofing granule production, USA	Renal diseases (ICD-10: N03–N05, N18, N19)	2650	1945–2004	1945–2004	772	16	1.76 (1.01–2.86)	0.9%	JEM (based mainly on expert judgements); 11.2
Reid and Sluis-Cremer (1996)	Gold mining, South Africa	Renal failure (ICD-9: 580–589)	4925	At work 1970	1970–1989	2032	24	1.64 (1.05–2.43)	1%	JEM, total duration of underground service, n/a
Steenland and Brown (1995)	Gold mining, South Dakota, USA	a)Acute renal disease (ICD-9: 580–581, 584), b) Chronic renal disease (ICD-9: 582–583, 585–587)	3328	1940–1975	1940–1990	1551	(a) 2 (b) 11	(a) 1.19 (0.14–4.29) (b) 1.25 (0.62–2.23)	2%	JEM; 9
Steenland and Sanderson (2001)	Sand industry, USA	a) Acute renal disease (ICD-9: 580–581, 584), b) Chronic renal disease (ICD-9: 582–583, 585–587) c) Registration of ESRD	4626	1960–1997	1960–1997	1073	(a) 3 (b) 10 (c) 23	(a) 3.37 (0.70–9.86) (b) 2.22 (1.06–4.08) (c) 1.97 (1.25–2.96)	n/a	JEM based on measurements 1974–1996, estimates for 1946 and interpolation; 9
Vacek et al. (2011)	Granite industry, Vermont, USA	Nephritis and nephrosis	7052	1947–1998	1947–2004	3831	34	0.99 (0.68–1.38)	1.25%	JEM based on measurements between 1924 and 2004; n/a

*n/a* not available

### Study characteristics

#### Industrial cohorts

The industrial cohort studies are rather homogeneous in their methodology. While mortality was used as the study outcome in most cohort studies, the investigators of two of the cohort studies were able to merge their databases with an end-stage renal disease (ESRD) registry. Hence, they could use incidence of ESRD and particularly GN as the study outcome (Steenland et al. 2001, 2002).

The included industrial cohort studies (Table 1) pertain to different industries, mainly sand and granite production. Many of the studies quantitatively estimated silica dust exposure for individual subjects with the aid of job-exposure matrices (JEM), but in many cases these were used only for internal analyses of the risk of lung cancer or non-malignant respiratory diseases. In seven studies, however, dose–response analyses were performed for renal diseases too. Where mentioned, studies with exposures to substances causing renal damage were excluded. As the focus of many studies was not on renal disease, information on relevant occupational co-exposures with respect to kidney is scarce. But for these diseases the use of mercury, for example, in gold mining in the Homestake Mine in South Dakota (Steenland and Brown 1995) may be relevant: The use of mercury there only ended in Dec 1970, when significant environmental damage was discovered in the region, especially in the waters (US Environmental Protection Agency RV 2007). However, among the industrial cohorts, there are several where relatively pure silica dust exposure can be assumed (Brown and Rushton 2005; McDonald et al. 2005; Vacek et al. 2011).

#### Silicosis registry cohorts

The ten cohort studies based on information from silicosis registries (Table 2) also investigated mainly mortality, though one study (Steenland et al. 2002) used registration with an ESRD registry as the outcome. In contrast to the industrial cohort studies, none of them estimated individual exposure to quartz dust. However, high levels of exposure are evident from the fact that subjects are drawn from silicosis registries, though allowances for individual susceptibility must be made. Mortality studies were geared toward assessing the risk of lung cancer or other respiratory diseases in conjunction with silica dust exposure. Therefore, they frequently excluded subjects with known exposure to other exposures (e.g., asbestos). However, exposure to lead, mercury, cadmium, or other substances known to damage the kidney was not discussed; hence, these occupational exposures cannot be ruled out.

**Table 2:** Cohort studies based on registries of silicotics

References	Geographical region	Outcome	Cohort size	Diagnostic period for silicosis	Follow-up period	Total deaths	Number of observed cases	Effect size (95%-CI)
Amandus et al. (1991)	North Carolina, USA	Mortality from chronic and unspecific failure of kidney (ICD8: 582–584)	760	1940–1983	1940–1983	550	2	1.4 (0.17–5.16)
Brown et al. (1997)	Sweden, Denmark	Mortality from urinary diseases	1130	SE: 1965–1883, DK: 1977–1989	SE: 1965–1989, DK: 1977-1990	795	9	1.6 (0.7–3.1)
Ebihara and Kawami (1998)	Japan	Mortality from nephritis and nephrosis (ICD9: 580–589)	850	1958–1995	1958–1995	599	5	1.13 (0.37–2.63)
Forastiere et al. (1989)	Latium, Italy	Mortality from genitourinary diseases (ICD-9: 580–629)	952	1946–1984	1969–1984	607	9	1.0 (0.46–1.9)
Marinaccio et al. (2006)	Tuscany, Italy	Mortality from nephritis, nephrotic syndrome, and nephrosis	14,929	1946–1979	1980–1999	8521	65	0.94 (0.73–1.20)
Ng et al. (1990)	Hongkong	Mortality from genitourinary diseases (ICD9: 580–629)	1419	1965–1981	1980–1986	356	2	0.49 (0.06–1.77)
Scarselli et al. (2011)	Latium, Italy	Mortality from nephritis, nephrotic syndrome, and nephrosis (ICD9: 580–589)	2034	1943–1986	1997–2006	1258	12	1.06 (0.55–1.86)
Starzynski et al. (1996)	Poland	Mortality from nephritis, nephrotic syndrome, and nephrosis (ICD9: 580–589)	11,935	1970–1985	1970–1991	3141	33	1.22 (0.84–1.71)
Steenland et al. (2002)	USA	(a) Registration of ESRD (b) glomerular nephropathy	1328	1984–1998	1984–1998	764	(a) 9 (b) 2	(a) 1.67 (0.76–3.17) (b) 4.19 (0.50–15.13)
Yu et al. (2007)	Hong Kong	Mortality from kidney disease (ICD9: 584–586)	2789	1981–1998	1981–1999	853	3	0.27 (0.05–0.78)

Many of the studies do not provide information on loss to follow-up or missing cause of death; however, silicosis patients have a much lower mobility than occupational cohorts and the recognition of silicosis as an occupational disease often entitles the patient to regular financial benefits. Missing information on loss to follow-up thus plausibly implies that the authors assume complete information. Similar considerations hold for cause of death information: Sweden and Poland have a national death index, and in Japan follow-up can be collected via the family register “Koseki”, such that complete information can be assumed for these three studies. The Italian studies, however, report missing cause of death information for two to nine percent of the deceased. As the time of entry into the studies equals the time of patients’ silicosis registration, previous renal diseases cannot be excluded and may even have led to some patients’ deaths before that point.

#### Case–control studies

The four case–control studies included here are heterogeneous in their choice of the outcome as well as in the source for selection of cases and controls and in the consideration of potential confounders in the risk assessment (Table 3).

**Table 3:** case–control studies

References	Geographical region	Study specification	Outcome	Number of subjects (cases/controls)	Period of recruitment	Response rates (among cases/among controls) %	OR (95%-CI)
Calvert et al. (2003)	27 states of USA	Individually matched study; five controls matched to each case based on sex, race, state of residence, five-year age group, and year of death group	Mortality: a) acute renal failure (ICD-9: 584), b) chronic renal failure (ICD-9: 585), d) all renal failure (ICD-9: 580–586), f) chronic glomerulonephritis (ICD-9: 581–582), h) membranous glomerulonephritis (ICD-9: 581.1, 582.1, 583.1)	a) 47,942/239,644 b) 56,521/282,492 d) 279,378/1,395,726 f) 12,563/62,804 h) (194/970)	1982–1995	100/100	a) 0.67 (0.32–1.39) b) 0.18 (0.06–0.56) d) 0.40 (0.28–0.57) f) 1.00 (0.22–4.56) h) 2.32 (0.2–25.63)
Chiazze et al. (1999)	US. glass wool production plants	Nested case–control study; controls matched by plant, year of birth (±2 *y*), and survival to the end of follow-up or death (±2 *y*)	Mortality; nephritis/nephrosis (ICD-9: 580–589): a) underlying cause b) underlying and contributing causes	a) 15/48 b) 47/172	Until 1994	100/100	a) 1.57 (0.07–46.73); b) 1.04 (0.24–4.46) for highest exposure category resp.
Steenland et al. (1990)	Michigan, USA	Controls pair-matched to cases by age, race, and area of residence	Incidence; selected diagnoses of ESRD (glomerulonephritis, nephrosclerosis, and interstitial kidney disease)	325/325	1976–1984	69/61	1.67 (1.02–2.74)
Vupputuri et al. (2012)	North Carolina, USA	Community controls are matched by age (±5 *y*), gender, race, and proximity to the hospital to hospital cases	Incidence of chronic renal disease (ICD-9: 250.4, 403, 404, 582–587, 590.0, 590.8, 593.9)	504/457	1980–1982	78/73	Chronic renal disease: 1.37 (1.02–1.85); Glomerulonephritis: 1.13 (0.63–2.03)

The assessment of exposure data may be particularly problematic in Calvert et al. (2003), where occupation and industry were ascertained from the information given on the death certificate. That information only existed for 2/3 of the subjects and is unreliable: When Bidulescu and colleagues examined the agreement between death certificate information and self-reported data concerning occupation, a complete match was derived only for 32% of deceased; coarser categories yielded Cohen’s *κ* = 0.6 (Bidulescu et al. 2007). Nevertheless, a clear positive trend in mortality odds ratios (MORs) with increasing exposure category was observed for silicosis, lung cancer, COPD, and pulmonary tuberculosis. In the highest exposure category, MOR = 30.5 was calculated for silicosis. Hence, this kind of exposure information seems to be suitable to roughly distinguish between highly and low exposed subjects. In contrast, even a negative trend was observed for chronic renal failure as well as for GN.

Investigating the influence of silica exposure on the risk of CKD development, major risk factors like diabetes and hypertension should be taken into account. Hence, an adjustment only for age and education as performed by Vupputuri and coworkers (Vupputuri et al. 2012) is not sufficient if a broad range of diagnoses for CKD are used. The descriptive data on cases and controls yield a crude OR = 6.22 for a history of diabetes and OR = 3.76 for daily use of analgesic. Hence, the actual contribution of silica to CKD risk remains unclear.

The study among employees in the fiberglass manufacturing industry did not show any noticeable risk increase with increasing exposure to respirable silica (Chiazze et al. 1999). However, the cumulative exposure was much lower than in quarrymen, for example. Only 11% of fiberglass workers have had a cumulative exposure of more than 0.5 mg/m^3^-years. In contrast, the highest quintile of granite workers has had a cumulative exposure of more than 6 mg/m^3^-years (Vacek et al. 2011). Hence, the cumulative exposure to respirable silica in fiberglass workers might be too low to lead to a detectable risk increase.

### Meta-analysis

While most mortality studies reported cases for the group ICD-9: 580-589 (nephritis, nephrotic syndrome, and nephrosis), some only provided information on the whole ICD-chapter X (diseases of the genitourinary system). From the few studies reporting both the group and the chapter mortality, it emerged that renal diseases constitute about 2/3 of cases in the chapter. Assuming that this proportion is constant, 1/3 of the observed and the expected cases were subtracted for those studies (Björ et al. 2013; Brown and Rushton 2005; Checkoway et al. 1997; Forastiere et al. 1989), forming the basis for comparative analyses of the industrial cohort studies and the silicosis registry cohort studies.

#### Meta-analysis of silicosis registry cohorts

**Fig. 2:**
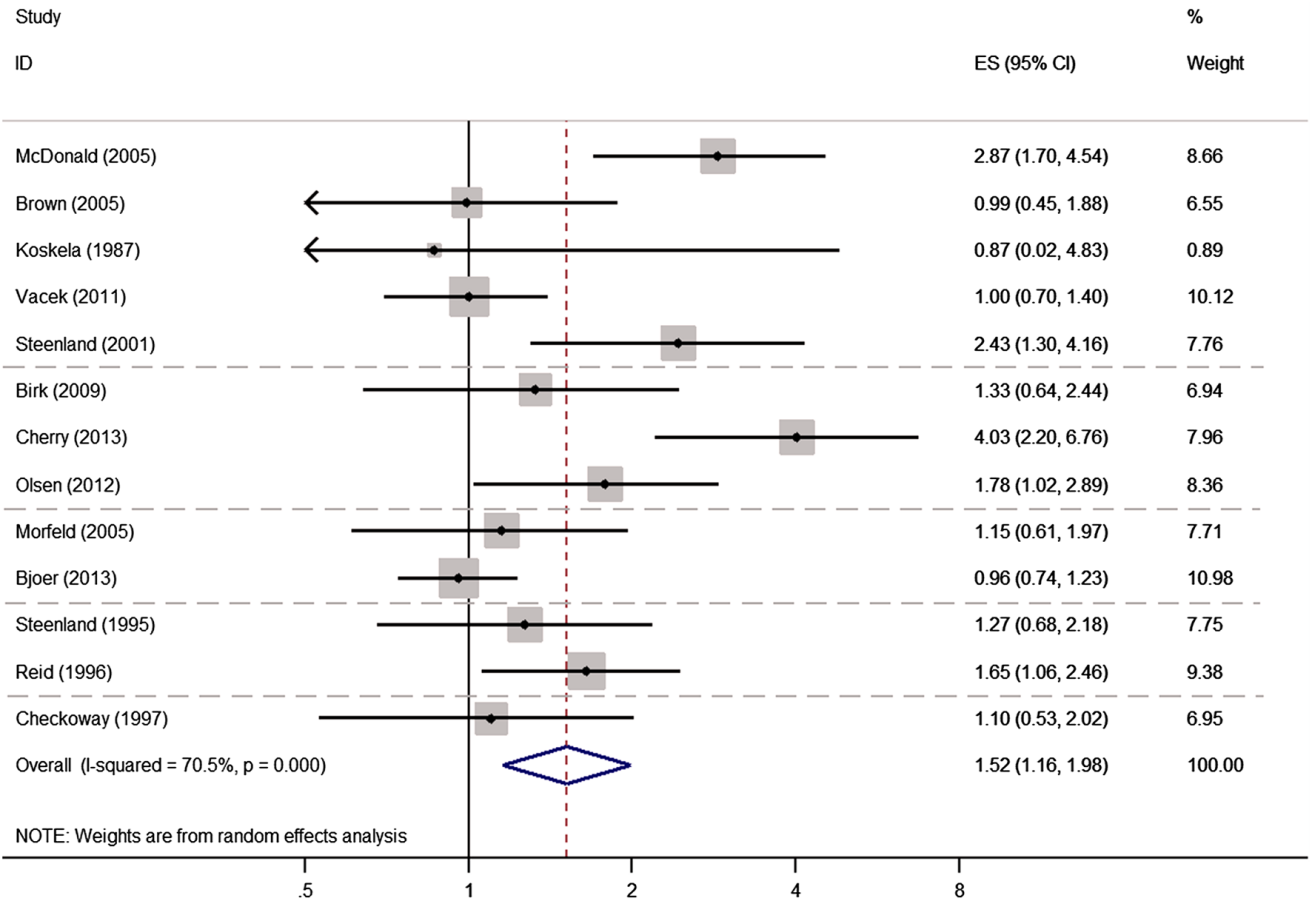
Meta-analysis of cohort studies on silicotics

The meta-analysis of the ten studies based on cohorts of silicotics applying a model with random effects yielded an overall SMR = 1.28 (95% CI 1.01–1.62), adjusted for competing risk of silicosis (Fig. 2). The proportion of variation in the SMRs due to heterogeneity between studies was 27.9%, based on I^2^. Here the Polish study was divided into two groups: in coal mining and other underground work, the SMR was close to the mean, while outside this industry it was almost twice as high, i.e., the ratio between the two SMRs was calculated to be 1.93 (95% CI 0.91–4.07).

#### Meta-analysis of industrial cohort studies

**Fig. 3:**
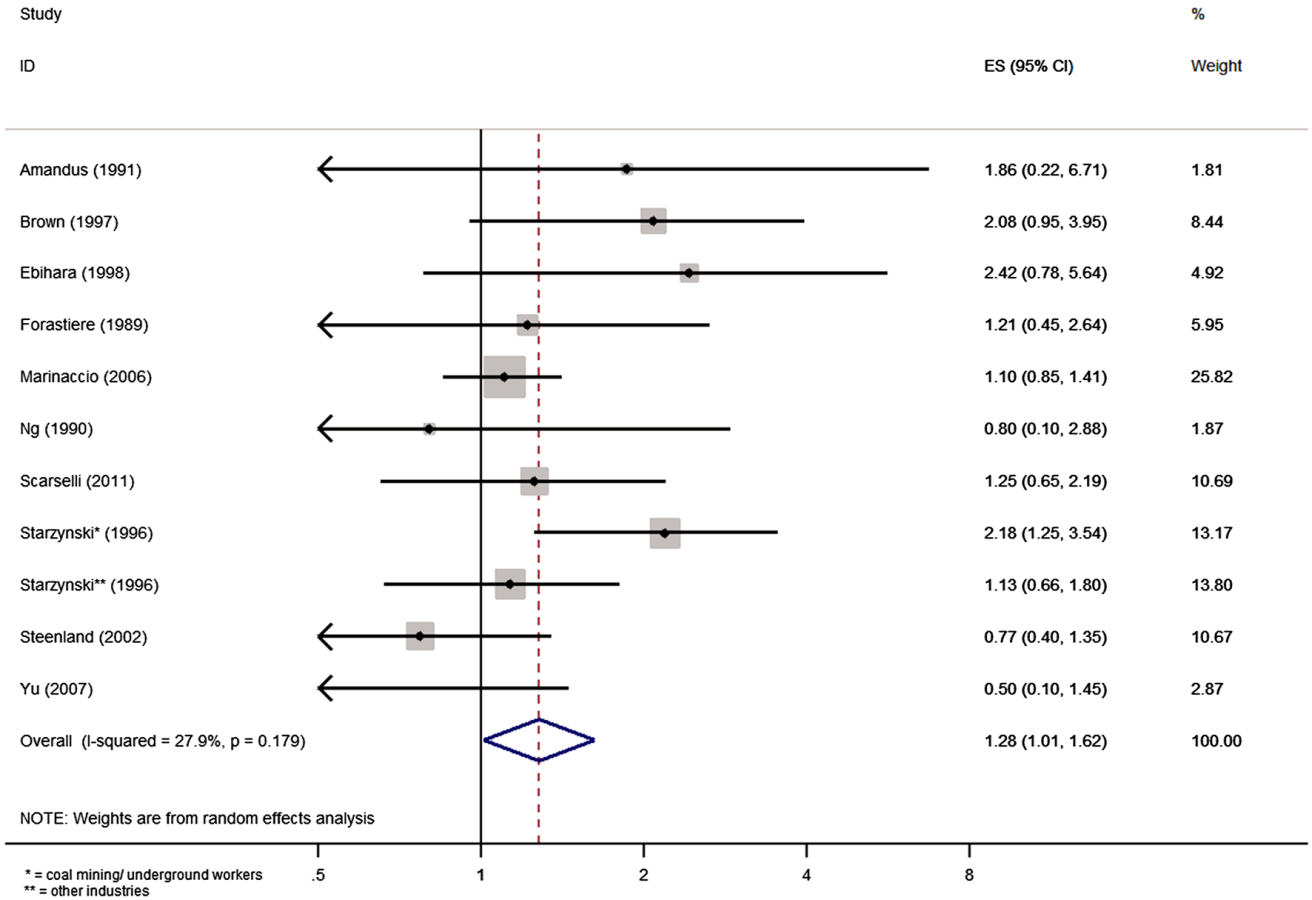
Meta-analysis of industry-based cohort studies

The combined analysis of the industry-based cohorts resulted in SMR = 1.52 (95% CI 1.16–1.98) (Fig. 3). However, the pronounced heterogeneity between these studies, leading to *I* ^2^ = 70.5%, required the use of a random effects model.

For the five sand and granite industry cohorts, the combined SMR (random effects model) was 1.59 (95% CI 0.91–2.78) with *I* ^2^ = 75.0%.

For the three pottery cohorts, the combined SMR (random effects model) was 2.15 (95% CI 1.13–4.08) with *I* ^2^ = 72.6%.

The two cohort studies from (coal and iron ore) mining industries had a combined SMR of 0.99 (95% CI 0.78–1.25).

The combined SMR of the two cohorts from gold mining was 1.51 (95% CI 1.07–2.12).

#### Dose–response analyses

In six of the industrial cohort studies, dose–response analyses were conducted for cumulative quartz dust exposure and renal disease. The results are heterogeneous: The study of US gold miners and one of the studies on sand workers (Steenland and Brown 1995; Steenland et al. 2001) showed significant positive dose–response associations between cumulative dust exposure and risk of renal disease mortality. The other four studies, however, did not find an increased risk for increased cumulative exposure (Cherry et al. 2013; McDonald et al. 2005; Olsen et al. 2012; Vacek et al. 2011). In one of them (McDonald et al. 2005), risk estimates for the higher exposure categories were as low as 0.2, though the negative trend could not be statistically verified owing to the small number of cases.

#### ESRD studies and GN sub-analyses

Glomerulonephritis as an outcome was considered in the industrial studies (Cherry et al. 2013; Steenland and Brown 1995; Steenland et al. 2001), reporting SIRs of 3.02 (*n* = 6), 4.24 (*n* = 5), and 3.85 (*n* = 7), in one silicosis registry study (Steenland et al. 2002) with an SIR of 2.65 (*n* = 4), and in three case–control studies (Calvert et al. 2003; Steenland et al. 1990; Vupputuri et al. 2012), reporting ORs of 1.67, 1, and 1.13 for silica dust exposure.

## Discussion

In recent decades, the focus of epidemiological studies on the health effects of silica was on cancer, especially on lung cancer, and on non-malignant respiratory diseases. First studies with a focus on the relationship between silica and non-malignant renal diseases were published only in the last quarter of the 20th century (Thun et al. 1982). The lack of information on possibly confounding factors for non-malignant renal diseases in the publications resulting from the late start of investigations concerning a possibly causal relationship is one of the reasons why this review emphasizes a more thorough discussion of various bias sources specific to the research question.

### Methodological issues

#### Search strategy

A common procedure for the selection of relevant studies for the review is to use the PICOS (participants, interventions, comparisons, outcomes, and study design) framework. It has already been demonstrated that it is usually more difficult to find optimal classifiers for the I and O elements than for the remaining elements (Boudin et al. 2010). Our first search string is closely based on the PICOS framework. The second one, however, has been chosen much broader in terms of the outcome variable, i.e., using the (general) mortality instead of some more detailed classifiers for renal diseases as the underlying cause of death. As the focus of many cohort studies was not on renal disease but on lung cancer or non-malignant respiratory disease, this approach reduced the risk to overlook cohort studies where SMR values for CRD appeared only in the tables but not in the abstract. Three registry-based and six industry-based cohort studies (30% of the primary studies included) were identified by the secondary search string only. These numbers underline the importance of the modified search strategy.

#### Publication bias

Related to the searching and finding of studies with CKD mortality is the issue of studies actually reporting it. There are indeed some large-scale occupational cohort studies not reporting mortality due to non-malignant kidney diseases (e.g., Chen et al. 2012). The probability of a study reporting CKD data may depend on the magnitude of the observed association, and thus a publication bias is conceivable.

#### Validity of the outcome

The primary goal of a study in occupational epidemiology is to identify risk factors in the occupational environment with an adverse impact on health; most often, the focus is on chronic diseases. But for a retrospective cohort study, which represents the most frequently used study design in this review, the outcome is usually defined on the basis of mortality data.

The temporal course from the onset of exposure to death can be divided into three phases. The duration of the first one, from the onset of exposure until the onset of disease, depends primarily on biological mechanisms and, if the exposure really has an adverse impact, on the extent of the exposure. The duration of the second phase, from the onset of disease until its diagnosis, largely depends on the general system of medical care, which includes among other aspects the health insurance system and occupational health screening, and also on the patient himself. The third phase is present only if the diagnosis is stated during the patient’s lifetime. The duration of this last phase, from diagnosis until death, is substantially driven by the severity of the disease and the medical care provided.

Etiological studies in occupational epidemiology aim at analyzing the impact of occupational exposures on the duration of the first phase only. Unfortunately, the individual time of disease onset is not known for most chronic diseases. Therefore, in general the time span between the time of first exposure and diagnosis (incidence data), i.e., the sum of phases one and two, should be analyzed.

For a retrospective mortality cohort study, the analysis is complicated by the additional variability of phase three: Up to a couple of years or even decades can elapse between diagnosis and death for diseases with a reasonably good prognosis, such as CKD. Go and colleagues have shown that patient’s survival is strongly correlated with the glomerular filtration rate at time of diagnosis (Go et al. 2004). Therefore, a periodically performed occupational health screening can help detect the disease already in an early, sometimes even in its preclinical stage by laboratory parameters, such as serum creatinine. Screening and diagnosis in the preclinical stage may have entailed an increase of the standardized incidence ratios (SIRs) for the occupational cohorts if the screening was not performed in the same manner in the reference population. On the other hand, an early diagnosis usually improves patient’s survival and, hence, reduces the SMR in mortality studies.

A further problem of using mortality data instead of incidence data is that the required population-based reference rates for the calculation of SMR are often available for the underlying cause of death only. Hence, the cause-specific incidence for the cause under investigation may be seriously underestimated, depending on the fatality rate of the disease, i.e., the larger the time span between first diagnosis and death, the lower the probability that the disease is assumed to be the underlying cause of death.

A much better approximation for the incidence can be achieved if the complete information from death certificates of study subjects is available as well as the multiple cause of death reference data (Steenland et al. 1992). From those death certificates, where nephritis, nephrotic syndrome, or nephrosis (N00–N07, N17–N19, N25–N27) was mentioned, it was documented as the underlying cause of death for less than 25%. But the difficulties for the analysis of the temporal correlation between exposure and outcome are the same as those for using the underlying cause of death as the outcome.

Lastly, when considering cause of death data for populations occupationally exposed to silica, one must bear in mind that an occupational disease such as silicosis may entail financial benefits for the dependents of the deceased. This may elevate the probability that it will be assigned a prominent place on the death certificate, thus suppressing other causes. On the other hand, the possibility of occupational diseases entails a higher probability of autopsy, making postmortem diagnoses of renal disease possible.

#### Outcome-specific sources of bias

CKD does not usually cause symptoms until it reaches an advanced stage. At earlier stages, it is usually detected by blood and urine tests. According to the 2003–2004 National Health and Nutrition Examination Survey, less than 5% of patients with stage 1 or 2 CKD and less than 10% with stage 3 reported having been diagnosed with CKD; only 45% of patients with stage 4 were aware of their condition (Plantinga et al. 2008). Moreover, health insurance and access to specialized medical care, i.e., a nephrologist, affects the time at which the disease is diagnosed for the first time (Ward et al. 2010).

Therefore, a considerable lead-time bias can be assumed if one compares a cohort of silica-exposed employees to the general population. These employees are mostly included in a surveillance program for silicosis [cf. (Steenland and Brown 1995; Steenland et al. 2001; Turner and Cherry 2000)] and they periodically undergo a thorough medical check-up. Consequently, a possible onset of a CKD can be diagnosed much earlier than in the reference population, resulting in an overestimation of the SIR.

Other issues of relevance for the estimation of risk become apparent in a study on pottery workers (Cherry et al. 2013): There, SMRs were computed for two reference populations, the national one and the regional one, resulting in strong differences in expected numbers. Expected numbers for overall mortality, silicosis, and lung cancer were much higher based on regional rates (Stoke-on-Trent) than based on national rates, indicating that the occupational exposure to quartz dust was much more common in the region than in England and Wales as a whole. Therefore, assuming that the incidence of CKD is positively correlated with exposure to quartz dust, one would also expect more cases based on regional rates in comparison to national rates. However, the region does not show an excess of renal disease: compared with the national mortality data, the expected numbers of cases are lower. The region may not always have had abundant nephrology health care, in fact it was one of the last to be included in the renal registry (Ansell et al. 2007); so the low expected numbers may also reflect less than optimal diagnostic conditions.

The SMR for renal disease is very high, however. One might conclude that even though silica dust did not influence renal mortality in the region, the cohort may have had better renal diagnostics, leading to an elevated SMR. Deaths in this cohort of pottery and other silica-exposed workers did indeed have regular statutory medical examination (Turner and Cherry 2000) and very high probability of being reported to a coroner for autopsy and adjudgement of cause of death (due to the possibility of occupational disease) (Meiklejohn 1949), which may have contributed considerably to the elevated SMR. Actually, this reasoning is confirmed by the study’s internal analysis, which observes no association between estimated exposures and renal disease.

For a more precise diagnosis of renal disease, such as glomerulonephritis (GN), a biopsy is often performed in early stages of disease to optimize the treatment. In advanced stages, the kidney will often be sclerotic, so that a biopsy would show nonspecific fibrotic changes only. Therefore, decreased renal size in ESRD is a contraindication for biopsy. On the other hand, especially for verifying the diagnosis such as GN a biopsy is needed (Fuiano et al. 2000). Hence, the incidence of GN is correlated with the biopsy rate in the catchment area (McQuarrie et al. 2009; Wirta et al. 2008). The incidence of GN consequently depends on pre-ESRD nephrologist care. The data of the U.S. Renal Data System clearly show that for patients without pre-ESRD nephrology care the incidence for GN was only half of that for patients with nephrology care for more than 12 months before ESRD (United States Renal Data System 2012). Therefore, a periodical medical check-up seems to lead on average to an earlier diagnosis and even to an apparent higher incidence for GN in comparison to the general population. This kind of diagnostic bias may have contributed to the observed higher standardized incidence ratio for GN in comparison to other ESRD in the US sand worker cohort (Steenland et al. 2001) as well as in the gold miner cohort (Steenland and Brown 1995).

#### Occupational co-exposures

The selection criteria ensured that cohorts with exposures to heavy metals (cadmium, lead, mercury, uranium, arsenic), which cause chronic renal disease with at least limited evidence, were already excluded, at least if they were mentioned in studies’ title, abstract, or keywords. Nevertheless, an exposure cannot be excluded categorically.

The industrial cohorts in this review pertain to different industries, where co-exposures may be present to different degrees. Additionally, one silicosis study (Starzynski et al. 1996) split the cohort of silicosis patients into four groups: coal miners; employees of underground work enterprises (drift cutting and shaft construction jobs); metallurgical industry and iron and non-ferrous foundry workers; and refractory materials, china, ceramics, and quarry workers. The separate analysis for underground workers, exposed mainly to silica and coal dust, yielded SMR = 1.13 (95% CI 0.66–1.81), whereas for the other job groups a significantly elevated risk was observed (SMR = 2.18, 95% CI 1.25–3.54). It can be assumed that rules concerning the recognition of occupational diseases as well as other health care conditions are identical for both groups. Hence, the difference between these two groups in terms of CKD risk seems to be related mainly to occupational co-exposures.

In gold mining, mercury is often used to amalgamate with gold. In the next step, the metal has to be recovered from the amalgam by boiling away the mercury. This refining procedure, which is hazardous due to the toxicity of mercury vapor, was also used in the gold mines investigated (Calvert et al. 1997; Reid and Sluis-Cremer 1996; Steenland and Brown 1995). Investigators assume that only 4 and 1% of the employees in the gold mine had the potential for exposure to lead and mercury, respectively (Calvert et al. 1997). The use of mercury in the refining process of the mine was not stopped until 1971, after studies of the US EPA had shown considerable environmental contamination by mercury (USEPA 2007). Hence, these co-exposures may have contributed to the observed elevated SMRs in the gold mining studies.

The cohorts from the pottery/ceramics industry (Birk et al. 2009; Cherry et al. 2013; Olsen et al. 2012) show elevated risks of renal diseases, and the SMRs are 1.32 (for men), 3.5, and 1.76. The Polish silicotics subcohort also yields a high SMR of 1.96. However, in the two studies with dose–response analyses, no significant trend was observed. Thus, the elevated risk may have been caused by other occupational exposures. For example, one co-exposure that was common in pottery work was lead. Indeed, lead poisoning in the glazing of earthenware was a common adverse health effect among exposed workers in the North Staffordshire potteries until the use of lead-containing glaze was forbidden by special regulations in 1947. Since then, a massive reduction of cases of lead poisoning was observed (Meiklejohn 1963). The cohort from the pottery industry comprised workers born between 1916 and 1945 who were hired from 1931 onwards (Cherry et al. 2013). So at least some of these workers could have been exposed to lead up to 20 years in their early working life. Given that chronic lead exposure is accumulated in the bones, the skeleton could serve as a repository of lead even after cessation of the exposure. Accumulated lead could be mobilized from this repository in later life by senile osteoporosis and target other organs, for example the kidneys (Hu et al. 1998). Especially in older people, often suffering from risk factors like diabetes, hypertension, or chronic kidney disease by other causes, the lead mobilized from the skeleton could contribute to nephrotoxicity, even at very low blood lead levels (Ekong et al. 2006), contributing to increased risk of renal disease mortality.

In coal mining, possible co-exposures include, albeit at a relatively low level, radon and its daughter products. Here, two cohorts from this industry were included (Morfeld et al. 2005; Starzynski et al. 1996). The German coal miners’ SMR for renal disease was only slightly elevated (1.13); the Polish coal workers’ SMR for renal disease was 0.94. Should the continuation of the large US (Attfield and Kuempel 2008) and UK (Miller and MacCalman 2010) coal miners studies yield similar effect estimates, no elevated risk of renal failure needs to be assumed for the coal mining industry. Even though the database for iron ore miners is much smaller than the one for coal miners, the results of the Swedish study (Björ et al. 2013) let us assume that this statement applies even to iron ore miners.

The sand and granite industries are the ones where silica dust exposure is fairly pure. Yet the results from these cohorts vary considerably. The studies from the US sand industry (McDonald et al. 2005; Steenland et al. 2001) report elevated risks (SMR 2.8 and 2.41), while the UK sand industry cohorts’ (Brown and Rushton 2005) SMR is 0.99. The two studies from the granite industry (Koskela et al. 1987; Vacek et al. 2011) report SMRs of 0.83 and 0.99.

#### Risk of bias

An overview of the risk of bias and the value for evidence for each of the studies is shown in Table 4. It should be pointed out that the classification is based on the assessment with respect to chronic non-malignant renal diseases only. However, in almost all cohort studies the focus was on lung cancer and/or chronic obstructive lung diseases. Therefore, our assessment does not reflect the quality of the study with respect to their primary research question.

**Table 4:** Assessment of risk of bias and studies’ value for evidence

Study	Bias due to					Study power	Statistical methods	Completeness of the reporting	Overall value for evidence
	Selection	Measurement of outcome	Exposure assessment	Confounding	Missing data				
Industrial cohorts									
Birk et al. (2009)	**	***	***	***	**	++	+++	+++	++
Björ et al. (2013)	****	**	***	***	***	+++	+++	++	+++
Brown and Rushton (2005)	****	**	****	***	***	++	+++	+++	++
Checkoway et al. (1997)	****	**	****	***	***	++	+++	+++	++
Cherry et al. (2013)	****	***	****	***	****	++	+++	++++	+++
Koskela et al. (1987)	****	***	**	***	***	+	++	+++	++
McDonald et al. (2005)	****	***	****	***	***	++	+++	+++	++
Morfeld et al. (2005)	**	***	****	***	****	++	+++	++++	++
Olsen et al. (2012)	****	***	****	***	****	++	+++	++++	++
Reid and Sluis-Cremer (1996)	**	***	**	**	****	++	++	++	++
Steenland and Brown (1995)	****	****	****	**	****	++	+++	+++	++
Steenland and Sanderson (2001)	***	****	****	**	***	++	+++	+++	+++
Vacek et al. (2011)	****	***	****	***	****	+++	++++	++++	+++
Cohorts of silicotics									
Amandus et al. (1991)	***	***	**	**	***	+	+++	+++	++
Brown et al. (1997)	***	**	**	**	***	++	+++	+++	++
Ebihara and Kawami (1998)	***	***	**	**	***	++	+++	+++	++
Forastiere et al. (1989)	***	**	**	**	***	++	+++	+++	++
Marinaccio et al. (2006)	***	***	**	**	***	+++	+++	+++	+++
Ng et al. (1990)	***	**	**	**	***	+	+++	+++	+
Scarselli et al. (2011)	***	***	**	**	***	++	+++	+++	++
Starzynski et al. (1996)	***	***	**	***	****	+++	+++	+++	+++
Steenland et al. (1992)	***	****	**	**	***	++	+++	+++	++
Yu et al. (2007)	***	***	***	**	***	+	+++	+++	+
Case–control studies									
Calvert et al. (2003)	****	****	**	**	**	++++	+++	+++	+++
Chiazze et al. (1999)	****	****	***	***	***	++	+++	+++	++
Steenland et al. (1990)	**	****	**	***	***	+++	+++	+++	++
Vupputuri et al. (2012)	***	****	**	***	***	++++	+++	+++	+++

* Critical risk, **** low risk

+ Low, ++++ high

### Non-occupational risk factors

Diabetes together with hypertension is the major cause of end-stage renal failure worldwide (Atkins 2005). On the other hand, these diseases are not only the major risk factors for CKD but also the major consequences of obesity and the metabolic syndrome. Furthermore, epidemiologic studies show that obesity and the metabolic syndrome are the independent risk factors for CKD. Nevertheless, it remains unclear in which way obesity or metabolic syndrome could directly harm the kidney (Wahba and Mak 2007). Discussed possible mechanisms involve inflammation related to insulin resistance, lipotoxicity, and hemodynamic effects by physical compression of the kidneys (Hall et al. 2014).

Thus, the general time trend in renal disease is connected to increasing incidence of diabetes: In aging populations, the prevalence of diabetes is rising and thus also the prevalence of its complications such as CKD (Menke et al. 2015).

One of the case–control studies examined the use of analgesics, confirming its association with CKD risks (Vupputuri et al. 2012). Unfortunately, this factor was not accounted for in any of the case–control studies.

### Glomerulonephritis

The proportion of renal failures caused by glomerulonephritis is difficult to estimate due to the diagnostic biases discussed above. Especially the lack of histologic information on death certificates makes GN difficult to detect. Recent data from German mortality statistics estimate it to be 1% of all deaths due to non-malignant kidney diseases (Statistisches Bundesamt 2014). Calvert and colleagues estimated it to be 4.5% (Calvert et al. 2003). Mortality studies are thus not well suited to examine GN. Incidence data from ESRD registers show that the proportion of GN in ESRD cases is about 10–20%, strongly depending on the prevalence of diabetes and the biopsy rate in the region.

The results from the GN studies in this review seem to show an increased risk of GN for workers exposed to respirable silica, but the observed numbers are very small and the possible biases, which mostly lead to overestimating the risk, are considerable. To draw conclusions on such a rare and difficult-to-detect outcome is therefore hardly possible without studies specifically targeting and monitoring GN.

## Conclusions

In the industrial cohorts, most SMRs are elevated and the silicotics cohort studies show the same picture, especially when using the competing risk-adjusting approach (Möhner 2016), giving the overall impression that workers exposed to respirable silica may indeed be at a higher risk of renal disease. However, the heterogeneity between studies is considerable and the sources of possible biases are plentiful.

In the cohort studies, the fact that most often mortality is the outcome under consideration blurs the incidence information. Moreover, in many industries, co-exposures that damage the kidney such as mercury or lead cannot be excluded; thus, in these cohorts they may have influenced the results.

Renal disease, when present, is not always diagnosed, so that the incidence of its reporting depends not only on the incidence of the disease itself, but also on the probability of its diagnosis. This in turn hinges on many external factors: the health insurance and health care system—if they differ systematically between cohort and reference population, then mortality patterns must be diverging. Statutory medical examinations of workers are one of the reasons for differences in diagnostic probabilities, and a probably higher autopsy rate among silica-exposed workers is another.

Therefore, even if the overall SMR for industry-based cohort studies as well as for cohorts of silicotics is significantly elevated, the higher risk cannot be attributed to respirable silica. Dose–response analyses may give a clearer picture. However, in this review, these have heterogeneous results and are often based on small numbers of cases; positive and negative trends are nearly in balance. This result is also in line with the results of a recent study on the relationship between silica exposure and renal disease or serum creatinine among silicotics, which was published outside of our time-window (Millerick-May et al. 2015).

In order to find the magnitude of a possible association, mortality studies are not optimal, even ESRD registry studies are not; instead, regular monitoring of early markers of renal disease such as creatinine should be established for a cohort in order to assess the incidence of renal disease. The U.S. National Kidney Foundation recommends three basic tests to screen for kidney disease: a quantitative test for protein or albumin in the urine (proteinuria), a calculation of glomerular filtration rate (GFR) based on a serum creatinine measurement, and a blood pressure measurement (National Kidney Foundation 2002). Biopsies could then even ascertain particular diagnoses such as GN.

The medical surveillance program developed by the U.S. National Industrial Sand Association could in the near future provide an ideally suited database for a renewed examination of a possible link between exposure to respirable silica and CKD—perhaps even with respect to GN (National Industrial Sand Association 2011).

## Electronic supplementary material

Below is the link to the electronic supplementary material.

Supplementary material 1 (DOCX 27 kb)
