# Supplementary material for: Occupational exposure to respirable crystalline silica and chronic non-malignant renal disease: systematic review and meta-analysis
Source: Int Arch Occup Environ Health. 2017 Apr 13;90(7):555–74. doi: 10.1007/s00420-017-1219-x (PMC5583269; doi:10.1007/s00420-017-1219-x)
Supplement: Supplementary file 1 — Supplementary material 1 (DOCX 27 kb) [file 420_2017_1219_MOESM1_ESM.docx]

### Occupational exposure to respirable crystalline silica and chronic non-malignant renal disease: systematic review and meta-analysis (Electronic supplementary material)

Authors and Affiliations

Matthias Möhner*, Anne Pohrt*, Johannes Gellissen*

* Federal Institute for Occupational Safety and Health, Berlin, Germany

Name and Address for Correspondence

Dr. Matthias Möhner

Federal Institute for Occupational Safety and Health

Division of work and health

Nöldnerstr. 40/42

10317 Berlin

Germany

Phone: +49 30 51548 4162

Fax: +49 30 51548 4170

E-mail: [Moehner.Matthias@baua.bund.de](mailto:Moehner.Matthias@baua.bund.de)

**Some information on excluded studies**

In the selection process for the present review many studies were excluded. On closer look some did not fulfil the inclusion criteria. Some studies were excluded that had been included in other Reviews on the association of quartz dust and renal disease, e.g. (Bartsch et al. 2004; Stratta et al. 2001). The reasons for their exclusion are discussed here.

A hospital-based case-control study with 16 cases and 32 controls is described in Gregorini et al. (1993). Cases were patients with ANCA (Antineutrophil cytoplasmic antibodies) with positive RPGN (rapidly progressive glomerulonephritis). Controls were patients from the same hospital department (nephrology), hence these were patients with renal disease. 12 of these controls were glomerulonephritis patients, such that the controls did not fulfil the criteria for our research question.

Wegener granulomatosis was the target disease in Nuyts et al. (1995). This systemic vasculitis is often associated with glomerulonephritis, especially with pauci-glomerulonephritis. 16 cases were examined in total. Population-based controls were matched by age, sex and geographic region. Five of the cases had been exposed to quartz dust; however, a number of other critical exposures were recorded among cases (7 chromium, 4 lead, 3 cadmium, 6 hydrocarbons). Due to the small number of cases, unadjusted odds ratios for the risk factors were reported. Hence a conclusion regarding the association of quartz dust exposure and risk of glomerulonephritis is impossible with this study.

The original cohort for Rapiti et al. (1999) were subjects from an Italian health surveillance programme installed specifically for ceramic workers. In this programme, elevated silicosis rates were recorded. The study determined prevalence of end-stage renal disease (ESRD) at a reference date (June 30, 1994) using an ESRD registry. It is thus a purely cross-sectional study, for which a diagnostic bias could be suspected. The use of prevalence data for occupational epidemiology must hence be considered with caution, which is why for the present review, only incidence and mortality studies were included. The study (Rapiti et al. 1999) had wrongly included the term “cohort study” in its keyword list, but we excluded it for the aforementioned reasons.

The authors in Hogan et al. (2001) describe two matched case-control studies, one on the association of ANCA associated small-vessel vasculitis (SVV), including 65 cases, and one on nephritis connected with systemic lupus erythematosus (SLE), including 51 cases with controls matched 1:1. The controls were recruited via a network of nephrologists, thus they, too, are patients with renal disease, which led to the exclusion of the study.

The study in Hogan et al. (2007) is again a matched case-control study similar to (Hogan et al. 2001), where cases had biopsy-proven glomerulonephritis as a result of ANCA associated SVV. In this study, however, recruiting population-based controls via random-digit dialling was attempted. In all, 129 cases and 109 controls were recruited, with response rates of 60% in cases and 12% in controls. The mean age of the cases was 7 years higher than that of controls. This discrepancy is hardly cancelled out by the crude age groups (18-39, 40-59, 60-84). So the methodological shortcomings as well as the deviating case definition led to the exclusion of this study.

The case-control study in Beaudreuil (2005) examines the occupational exposures of ANCA positive patients. In all, 60 cases are considered, of which only 10 have renal diseases. However, there were no separate analyses for the entities. Additionally, response rates are missing for cases as well as controls.

References

Bartsch R, Stein G, Schiele R (2004) Quarzstaubexposition und Nierenerkrankungen. Arbeitsmed Sozialmed Umweltmed 39(11)

Beaudreuil (2005) Occupational exposure in ANCA-positive patients: A case-control study. Kidney International 67:1961-1966

Gregorini G, Ferioli A, Donato F, Tira P, Morassi L, Tardanico R, Lancini L, Maiorca R (1993) Association between silica exposure and necrotizing crescentic glomerulonephritis with p-ANCA and anti-MPO antibodies: a hospital-based case-control study. AdvExpMedBiol 336:435-440

Hogan SL, Cooper GS, Savitz DA, Nylander-French LA, Parks CG, Chin H, Jennette CE, Lionaki S, Jennette JC, Falk RJ (2007) Association of silica exposure with anti-neutrophil cytoplasmic autoantibody small-vessel vasculitis: a population-based, case-control study. Clinical journal of the American Society of Nephrology : CJASN 2(2):290-299 doi:CJN.03501006 [pii];10.2215/CJN.03501006 [doi]

Hogan SL, Satterly KK, Dooley MA, Nachman PH, Jennette JC, Falk RJ (2001) Silica exposure in anti-neutrophil cytoplasmic autoantibody-associated glomerulonephritis and lupus nephritis. JAmSocNephrol 12(1):134-142

Nuyts GD, Van VE, De VA, Daelemans RA, Rorive G, Elseviers MM, Schurgers M, Segaert M, D'Haese PC, De Broe ME (1995) Wegener granulomatosis is associated to exposure to silicon compounds: a case-control study. NephrolDialTransplant 10(7):1162-1165

Rapiti E, Sperati A, Miceli M, Forastiere F, Di LD, Cavariani F, Goldsmith DF, Perucci CA (1999) End stage renal disease among ceramic workers exposed to silica. OccupEnvironMed 56(8):559-561

Stratta P, Messuerotti A, Canavese C, Coen M, Luccoli L, Bussolati B, Giorda L, Malavenda P, Cacciabue M, Bugiani M, Bo M, Ventura M, Camussi G, Fubini B (2001) The role of metals in autoimmune vasculitis: epidemiological and pathogenic study. SciTotal Environ 270(1-3):179-190
